# Effects of lymph node metastasis of thoracic esophageal squamous cell carcinoma on design of radiotherapy target volume

**DOI:** 10.12669/pjms.35.1.431

**Published:** 2019

**Authors:** Guobing Pan, Haitao Pan, Yuang Zhang, Haitao Shuai

**Affiliations:** 1 Guobing Pan, Department of Medical Engineering, The 476th Hospital of PLA, Fuzhou 350002, Fujian Province, P. R. China; 2 Haitao Pan, Department of Radiology, The 476th Hospital of PLA, Fuzhou 350002, Fujian Province, P. R. China; 3 Yuang Zhang, Department of Radiotherapy, The 476th Hospital of PLA, Fuzhou 350002, Fujian Province, P. R. China; 4 Haitao Shuai, Department of Radiology, The 476th Hospital of PLA, Fuzhou 350002, Fujian Province, P. R. China

**Keywords:** Esophageal squamous cell carcinoma, Lymph node metastasis, Radiotherapy

## Abstract

**Objective:**

To analyze the correlation between lymph node metastasis of thoracic esophageal squamous cell carcinoma (ESCC) and clinical and pathological factors, and to provide a reference for the outline of clinical target volume.

**Methods:**

The pathological characteristics of 1034 thoracic ESCC patients after surgery were described, and the correlations between clinical and pathological factors and lymph node metastasis were studied by univariate and Logistic multivariate analyses.

**Results:**

Lymph node metastasis was significantly correlated with tumor length, invasion depth and differentiation degree (P<0.01), but not gender, age, tumor site or pathological type (P>0.05). Logistic multivariate analysis showed that tumor length, invasion depth and differentiation degree were independent risk factors for thoracic ESCC. The lymph node metastasis rates of mid-thoracic ESCC in the middle mediastinum, lower-thoracic ESCC in the lower mediastinum and abdominal cavity were 18.5%, 35.3% and 19.7% respectively in the T1-T2 stage. In the T3-T4 stage, the lymph node metastasis rates of mid-thoracic ESCC in the middle mediastinum and abdominal cavity were 39.6% and 17.4% respectively, and those of lower-thoracic ESCC in middle and lower mediastina and abdominal cavity were 21.1%, 43.4% and 29.8% respectively. Highly/moderately differentiated mid-thoracic ESCC in the middle mediastinum, lower-thoracic ESCC in middle and lower mediastina and abdominal cavity had the lymph node metastasis rates of 34.7%, 15.1%, 33.5% and 23.7% respectively. Lowly differentiated mid-thoracic ESCC in the middle mediastinum and abdominal cavity had the lymph node metastasis rates of 46.9% a 29.6% respectively, and those of lower-thoracic ESCC in middle and lower mediastina and abdominal cavity were 25.5%, 49.1% and 27.3% respectively.

**Conclusion:**

During the outline of radiotherapy target volume for thoracic ESCC, tumor length, invasion depth and differentiation degree should be comprehensively considered to selectively irradiate the regions prone to lymph node metastasis.

## INTRODUCTION

Esophageal cancer prevails in China, with the morbidity and morbidity rates ranking fourth among those of all malignant tumors.^1^ Although surgery, radiotherapy and chemotherapy have been widely used, the 5-year survival rates of patients are still as low as 10%-30%.^2^ Most patients have entered the middle and advanced stages upon diagnosis, so the prognosis is mostly affected by postoperative recurrence and lymph node metastasis.^3^

Recently, researchers have endeavored to determine the TNM stage of esophageal cancer and the rational range of lymph node dissection, as well as to outline the target volume for accurate postoperative radiotherapy. Particularly, the state of lymph node evidently affects TNM stage, design of treatment regimen and evaluation of prognosis.^4^ On one hand, the radiotherapy for esophageal cancer is used to improve the dose distribution of tissues with pathological changes and peripheral normal ones together with organs at risk by using physical means, thereby increasing the local control rate. On the other hand, the radiotherapy target volume can be accurately regulated.^5^ The therapeutic effects of 3D-CRT and routine radiotherapy have been compared.^6^,^7^ The former is markedly superior to the latter in the dose distribution of target volume and the risk to peripheral normal tissues. 3D-CRT manages to overcome the disadvantages induced by large T-shaped field of postoperative prophylactic radiotherapy, such as high tumor residual rate because of unsatisfactory dose distribution.

Accurate determination of the target volume range plays a key role in 3D-CRT. For the radiotherapy of esophageal cancer, accurate outline of the target volume for lymphatic drainage is of great significance to an entire treatment plan, which can prolong the survival and improve the quality of life of patients. Postoperative prophylactic radiotherapy for esophageal cancer cannot elevate the survival rate, but inhibit lymph node metastasis and raise the local control rate.^8^ In this study, we retrospectively analyzed the clinical data of 1034 patients with thoracic esophageal squamous cell carcinoma (ESCC), aiming to clarify the clinical and pathological factors affecting lymph node metastasis, and to provide valuable clinical evidence for designing the radiotherapy target volume.

## METHODS

### Case selection and baseline clinical data

This study has been approved by the ethics committee of our hospital, and written consent has been obtained from all patients. A total of 1034 patients with thoracic ESCC receiving surgery in our hospital from October 2006 to October 2017 were selected, consisting of 804 males and 230 females aged 30-85 years old (median: 61). All patients did not receive radiotherapy or chemotherapy before surgery. Besides, 47, 688 and 299 cases had upper-thoracic, mid-thoracic and lower-thoracic tumors respectively. The tumor length was (4.3 ± 1.8) cm. According to the 2002 AJCC TNM staging criteria for esophageal cancer, there were 6, 59, 186, 639 and 144 cases in T_is_, T1, T2, T3 and T4 stages respectively. There were 233, 649 and 152 cases of highly, moderately and lowly differentiated cases respectively (Table-I).

**Table-I:** Logistic univariate analysis of risk factors for thoracic ESCC metastasis (n)

Factor	n	Lymph node metastasis	χ^2^	P
Gender			0.003	0.959
Male	804	369		
Female	230	106		
Age (year)			2.603	0.272
40	11	6		
40~60	494	241		
>60	529	233		
Tumor site			5.543	0.063
Upper-thoracic	47	29		
Mid-thoracic	688	307		
Lower-thoracic	299	143		
Pathological type			0.617	0.439
Ulcerative	520	241		
Medullary	415	191		
Fungating	72	31		
Constrictive	22	10		
Intralumenal	5	2	
Tumor length (cm)			22.314	0.01
2.0	136	55		
2.0~4.0	442	176		
4.0~6.0	336	177		
6.0~8.0	91	51		
>8.0	29	19		
Invasion depth			12.006	<0.01
T_is_	6	4		
T1	59	18		
T2	185	82		
T3	639	289		
T4	145	80		
Differentiation degree			9.405	<0.01
High	232	92		
Moderate	649	296		
Low	153	85		

### Surgical Methods

All patients received routine ultrasound or CT examination before surgery. If the cervical lymph node did not swell (short diameter >0.5 cm), incisions were made in the middle of the right thorax and upper abdomen to perform subtotal esophagectomy and standard two-field lymph node dissection. If the cervical lymph node swelled, U-shaped incisions were made through the right thorax, upper abdomen and both sides of the neck to carry out three-field lymph node dissection. The dissected lymph nodes of each site were examined by Department of Pathology in our hospital. After surgery, 400 patients did not receive chemotherapy or radiotherapy, and 134 patients were subjected to 1-6 weeks of chemotherapy using cisplatin and 5-fluorouracil. Additionally, 500 patients received radiotherapy, of whom 350 and 150 were given three-dimensional conformal radiotherapy and two-dimensional radiotherapy respectively. The three-dimensional conformal radiotherapy covered the esophageal tumor bed and corresponding mediastinal lymph node drainage areas, while the two-dimensional radiotherapy involved the esophageal tumor bed and total mediastinal lymph node drainage area, both using 6 MV X-ray linear accelerator. Meanwhile, of the 500 patients, 120 received ^60^Co radiation at bilateral supraclavicular lymph node drainage areas with anterior and posterior two-field radiation (30-36 Gy) in the early course and bilateral longitudinal or anterior oblique radiation (45-55 Gy) in the late course. For the patients with residual positivity, central radiation at 60 Gy was performed. They were radiated through routine fractionation once daily, 1.8-2.0 Gy each time and five times each week. The mediastinal radiation field had a width of 6-8 cm and a length of 15-27 cm.

### Grouping of Lymph Nodes

Lymph nodes were grouped and numbered according to the revised criteria of Japanese Society for Esophageal Diseases.^9^ Cervical lymph nodes were divided into 101, 102, 103 and 104 groups, lymph nodes in the upper mediastinum were divided into 105, 106rec, 106pre and 106tb groups, those in the middle mediastinum were divided into 107, 108, 109 and 112 groups, those in the lower mediastinum were divided into 110 and 111 groups, and those in the abdominal cavity were divided into 1~11 groups.

### Statistical Analysis

All data were analyzed by SPSS18.0. The correlations between clinical and pathological factors and lymph node metastasis were subjected to the χ^2^ test. Logistic multivariate analysis was conducted for the significant factors in univariate analysis by using the forward stepwise regression method. P<0.05 was considered statistically significant.

## RESULTS

Lymph node metastasis was significantly correlated with tumor length (χ^2^ = 22.314, P<0.01), invasion depth (χ^2^ = 12.006, P<0.01) and differentiation degree (χ^2^ = 9.405, P<0.01), but not gender, age, tumor site or pathological type (P>0.05) (Table-I). Logistic multivariate analysis showed that tumor length, invasion depth and differentiation degree were independent risk factors for thoracic ESCC metastasis (Table-II).

**Table-II:** Logistic multivariate analysis of risk factors for thoracic ESCC metastasis

Factor	Coefficient of regression	Standard error	Wald value	P	OR	95%CI
Differentiation degree	0.312	0.074	17.886	<0.01	1.366	1.182~1.579
Tumor length	0.292	0.049	35.703	<0.01	1.339	1.217~1.474
Invasion depth	0.226	0.060	14.301	<0.01	1.253	1.115~1.409

The lymph node metastasis rate of upper-thoracic ESCC into the upper mediastinum was 34.0% which was significantly higher than those of mid- (6.5%) and lower-thoracic tumors (3.3%) (P<0.05). The lymph node metastasis rate of lower-thoracic ESCC into the lower mediastinum was 41.5% which significantly exceeded those of mid- (4.7%) and upper-thoracic tumors (12.8) (P<0.05). Mid-thoracic ESCC was prone to metastasis into lymph nodes of both the middle mediastinum (36.5%) and abdominal cavity (18.2%) (Table-III).

**Table-III:** Lymph node metastasis rates of upper-thoracic, mid-thoracic and lower-thoracic ESCC [n (%)].

Site	n	Cervical	Upper mediastinum	Middle mediastinum	Lower mediastinum	Abdominal cavity
Upper-thoracic	47	7 (14.9)	16 (34.0)∆,**	8 (17.0)	6 (12.8)**	5 (10.6)
Mid-thoracic	688	31 (4.5)	45 (6.5)∆	251 (36.5)	32 (4.7)*	125 (18.2)
Lower-thoracic	299	7 (2.3)	10 (3.3)∆,**	58 (19.4)	124 (41.5)**	83 (27.8)

Total	1034	45 (4.4)	71 (6.9)	317 (30.7)	162 (15.7)	213 (20.6)

Compared with mid- and lower-thoracic ESCC,

* P<0.01; compared with upper- and lower-thoracic ESCC,

** P<0.01; compared with upper- and mid-thoracic ESCC, ∆P<0.01.

The lymph node metastasis rates of mid-thoracic ESCC in the middle mediastinum, lower-thoracic ESCC in the lower mediastinum and abdominal cavity were 18.5%, 35.3% and 19.7% respectively in the T1-T2 stage. In the T3-T4 stage, the lymph node metastasis rates of mid-thoracic ESCC in the middle mediastinum and abdominal cavity were 39.6% and 17.4% respectively, and those of lower-thoracic ESCC in middle and lower mediastina and abdominal cavity were 21.1%, 43.4% and 29.8% respectively. Highly/moderately differentiated mid-thoracic ESCC in the middle mediastinum, lower-thoracic ESCC in middle and lower mediastina and abdominal cavity had the lymph node metastasis rates of 34.7%, 15.1%, 33.5% and 23.7% respectively. Lowly differentiated mid-thoracic ESCC in the middle mediastinum and abdominal cavity had the lymph node metastasis rates of 46.9% a 29.6% respectively, and those of lower-thoracic ESCC in middle and lower mediastina and abdominal cavity were 25.5%, 49.1% and 27.3% respectively. With increasing length of mid-thoracic ESCC, the lymph node metastasis into the middle mediastinum rose. The lymph node metastasis rate of lower-thoracic ESCC into the lower mediastinum was similar to that mid-thoracic ESCC (Table-IV).

**Table-IV:** Lymph node metastasis of mid- and lower-thoracic ESCC with different pathological factors.

Site	n	Cervical	Upper mediastinum	Middle mediastinum	Lower mediastinum	Abdominal cavity
*Invasion depth*						
T1-T2						
Mid-thoracic	173	5 (2.9)	13 (7.5)	32 (18.5)	8 (4.6)	18 (10.4)
Lower-thoracic	71	1 (1.4)	2 (2.8)	10 (14.1)	25 (35.2)	14 (19.7)
T3-T4						
Mid-thoracic	556	25 (4.5)	33 (5.9)	220 (39.6)	25 (4.5)	97 (17.4)
Lower-thoracic	228	6 (2.6)	8 (3.5)	48 (21.1)	99 (43.4)	68 (29.8)
*Differentiation degree*						
High to moderate						
Mid-thoracic	590	25 (4.2)	37 (6.3)	205 (34.7)	23 (3.9)	87 (14.7)
Lower-thoracic	291	6 (2.1)	10 (3.4)	44 (15.1)	96 (33.0)	69 (23.7)
Low						
Mid-thoracic	98	5 (5.1)	9 (9.2)	46 (46.9)	10 (10.2)	29 (29.6)
Lower-thoracic	55	1 (1.8)	0 (0)	14 (25.5)	27 (49.1)	15 (27.3)
*Tumor length (cm)*						
<4						
Mid-thoracic	413	12 (2.9)	18 (4.4)	109 (26.4)	10 (2.4)	44 (10.7)
Lower-thoracic	165	3 (1.8)	3 (1.8)	27 (16.4)	54 (32.7)	40 (24.2)
4~6						
Mid-thoracic	224	14 (6.3)	17 (7.6)	95 (42.4)	13 (5.8)	48 (21.4)
Lower-thoracic	112	3 (2.7)	5 (4.5)	21 (18.8)	47 (42.0)	28 (25.0)
>6						
Mid-thoracic	81	5 (6.2)	10 (12.3)	47 (58.0)	9 (11.1)	23 (28.4)
Lower-thoracic	39	1 (2.6)	2 (5.1)	11 (28.2)	22 (56.4)	14 (35.9)

## DISCUSSION

Lymph node metastasis is the most common route of metastasis of esophageal cancer, of which squamous carcinoma accounts for 95%, with the main site of occurrence in the thoracic esophagus.^10^ Therefore, this study retrospectively analyzed the lymph node metastasis pattern and the impact of clinical pathological factors on lymphatic metastasis in 1034 cases of ESCC patients undergoing surgery, which has important guiding significance for the development of a reasonable radiation field. The eradication of three-field lymph nodes for thoracic ESCC can improve the accuracy of pathological staging and reduce the local recurrence rate.^11^ However, this type of surgery has a high postoperative complication and its value has long been debated. During the surgery of thoracic ESCC in our hospital, preoperative ultrasound scans or CT disclosed that the patients with cervical lymph node metastasis for selective three-field lymph node clearance could not only ensure the thorough removal of lymph nodes, but also effectively reduce the risk of surgery.

Upper-thoracic ESCC mainly metastasized to the neck and upper and middle mediastinal lymph nodes, and the extent of its downward mediastinal and abdominal lymph node metastasis decreased. The metastasis of lymph nodes in the mid-thoracic ESCC was bilateral, and the lymph nodes of the lower-thoracic ESCC metastasized mainly to the medial and lower mediastinal and abdominal lymph nodes.^12^ We found that the site of lymph node metastasis was different in each segment of thoracic ESCC. For upper-thoracic ESCC, the cervical lymph node metastasis and the upper mediastinal metastasis accounted for 14.9% and 34.0%, respectively. The mid-thoracic ESCC showed the bidirectional metastatic tendency. The upper mediastinal, cervical, lower mediastinal and abdominal lymph node metastasis accounted for 6.5%, 4.5%, 4.7% and 18.2%, respectively. The lower-thoracic ESCC mainly metastasized to lower mediastinum (41.5%) and abdominal lymph nodes (27.8%), and cervical lymph node metastasis was rarely seen, which is roughly the same as reported in the above literature.

Thoracic ESCC is preferred to choose surgery, but many patients cannot be treated surgically because of distant metastasis or high risks.^13^ For locally advanced and inoperable patients, the role of radiotherapy has become increasingly prominent. Due to the uncertainty of lymph node metastasis, the accurate delineation of clinical target areas has become the major bottleneck of radiotherapy. Accurately defining the irradiated target area becomes the focus and difficulty of current radiotherapy. It is clearly stipulated in RTOG85-01^14^ and RTOG94-05^15^ that 3-5cm away from the upper and lower ends of tumor is determined as clinical target areas, but subclinical infiltration of lymph nodes is not fully considered. Based on the results herein, we recommend that the target area of T1-T2 mid-thoracic ESCC radiotherapy only includes lymph node drainage area in the mediastinal region, and that of the lower-thoracic region includes only the mediastinal and abdominal lymphatic drainage regions; while T3-T4 mid-thoracic ESCC target areas include lymph node drainage areas in the mediastinal and abdominal regions, and those in the lower-thoracic region includes the lymph node drainage areas in the mediastinum, lower mediastinum and abdominal area. The target area of high-medium-differentiated mid-thoracic ESCC radiotherapy includes only the mid-mediastinal lymph node drainage region, and that of high-medium-differentiated lower-thoracic ESCC radiotherapy includes the lymph node drainage area in the middle and lower mediastinal and abdominal regions; and the radiotherapy target area of poorly differentiated mid-thoracic esophagus squamous carcinoma includes the mid-mediastinal and abdominal lymphatic drainage area, and that of poorly differentiated lower-thoracic ESCC includes the lymph node drainage area of the middle and lower mediastinal and abdominal areas. The radiotherapy target area of the mid-thoracic ESCC with a tumor length <4 cm includes only the mid-mediastinal lymph node drainage area, and that of lower-thoracic ESCC with a tumor length <4 cm includes the middle and lower mediastinal and abdominal lymphatic drainage areas; the radiotherapy target area of mid-thoracic ESCC with a tumor length of 4-6 cm includes the mid-mediastinal and abdominal lymph node drainage area, and that of lower-thoracic ESCC with a tumor length of 4-6 cm includes middle and lower mediastinal and abdominal lymph node drainage areas; the radiotherapy target area of mid-thoracic ESCC with a tumor length of >6 cm includes mid-mediastinal and abdominal lymph node drainage area, and that of lower-thoracic ESCC with a tumor length of >6 cm includes the middle and lower mediastinal and abdominal lymph node drainage areas.

In summary, when radiotherapy target area of thoracic ESCC is delineated, ultrasound, CT, depth of tumor infiltration, differentiation degree and tumor length should be considered comprehensively to selectively irradiate high-risk areas of lymph node metastasis, which can both ensure the accuracy of clinical and subclinical target area irradiation, and reduce the risk of radiotherapy so as to improve the local control rate and overall survival.

### Authors’ contributions

**GP & HP:** Designed this study and revised this manuscript.

**YZ & HS:** Performed this study and drafted this manuscript.
